# Psychometric Performance of the Cannabis Use Disorders Identification Test– Revised (CUDIT-R) in an Youth Clinical Sample

**DOI:** 10.1007/s10597-025-01494-5

**Published:** 2025-07-23

**Authors:** Alice Palmer, Simon Adamson, Ria Schroder, Lisa Wood

**Affiliations:** 1 Division of Psychiatry, University College London, Maple House, 149 Tottenham Court Road, London, W1T 7NF UK; 2 University of Otago, Dunedin, New Zealand

**Keywords:** Youth, Cannabis, Cannabis use disorder, Screening

## Abstract

The Cannabis Use Disorders Identification Test–Revised (CUDIT-R) is an eight-item screening tool designed to identify problematic cannabis use. The present study intended to assess the psychometric properties of the measure in a youth sample. *N* = 76 participants completed the CUDIT-R and a measure of cannabis consumption. All participants underwent an assessment for a current cannabis use disorder using the Structured Clinical Interview for DSM-IV. The CUDIT-R exhibited good internal consistency (Cronbach’s α = 0.76) and concurrent validity with a cannabis consumption measure. Discriminant validity was demonstrated with an area under the ROC curve of 0.96. The CUDIT-R proved to be a reliable and valid screening tool amongst youth with heavy cannabis use, which suggests the scale has clinical utility.

**Supplementary Information:**

The online version contains supplementary material available at 10.1007/s10597-025-01494-5.

## Introduction

Cannabis is the third most commonly used psychoactive substance globally, following alcohol and tobacco. In 2019, an estimated 200 million individuals aged 15–64 reported using cannabis at least once, with usage rates peaking among youth aged 15–16 (UNODC, 2018, 2021). Over the past decade, the legal and cultural landscapes surrounding cannabis have shifted significantly, with over 20 countries legalising its medical use and numerous others decriminalising recreational consumption (Pacula & Smart, 2017). These regulatory changes have coincided with a decline in the perceived risks associated with cannabis use, particularly among youth (UNODC, 2018; Johnston et al., 2019).

This trend is particularly concerning, as youth—typically defined as individuals aged 10–19—may be more susceptible to the adverse consequences of cannabis use compared to adults (World Health Organization, 2019). Empirical evidence links youth cannabis use to a range of short- and long-term negative outcomes, including respiratory problems (Tashkin et al., 2002), diminished educational attainment (Silins et al., 2015), and reduced life satisfaction (Fergusson & Boden, 2008). Furthermore, cannabis use during adolescence is associated with increased risk for a spectrum of psychiatric disorders, including psychotic, mood, and substance use disorders, in a dose-dependent manner (Levine et al., 2017). The most robust association exists between youth cannabis use and the development of psychotic disorders, particularly with frequent use of high-potency tetrahydrocannabinol (THC) strains (Malone et al., 2010; Di Forti et al., 2019). Additionally, a recent meta-analysis found a modest but significant association between youth cannabis use and increased risk of depression, self-harm, and suicidal behaviours (Gobbi et al., 2019).

The frequency and intensity of cannabis use further compound these risks. Early initiation and regular use have been consistently linked with the development of cannabis use disorder (CUD), a condition defined by the persistent use of cannabis despite significant distress or impairment in functioning (American Psychiatric Association [APA], 2013). A recent meta-analysis estimated that approximately one in six young people who use cannabis develop CUD, with this rate rising to nearly one in three in those who use weekly (Leung et al., 2020).

Given the high prevalence of cannabis use and the serious associated outcomes among youth, there is a pressing need for reliable and valid screening tools to identify problematic cannabis use in this population. Effective screening instruments enable clinicians to detect harmful patterns of use, assess severity, and monitor treatment outcomes (Bonn-Miller et al., 2016; Adamson et al., 2014). While several tools exist for adult populations, relatively little research has focused on the development or validation of such instruments for youth (Annaheim & Legleye, 2017).

One of the most widely used screening instruments for cannabis use among adults is the Cannabis Use Disorders Identification Test-Revised (CUDIT-R; Adamson et al., 2010). This eight-item, self-report questionnaire assesses problematic cannabis use over the past six months, capturing domains such as consumption, dependence, and psychological consequences. Responses are scored on a five-point Likert scale, with total scores ranging from 0 to 32. Higher scores indicate more severe use, and specific cut-offs are used to guide further assessment (Adamson et al., 2010).

The CUDIT-R has demonstrated solid psychometric properties in adult populations, including strong internal consistency (e.g., Cronbach’s α = 0.88 in a college student sample; Mezquita et al., 2022) and predictive validity with established measures such as the cannabis use scale of the Opiate Treatment Index (Spearman’s ρ = 0.662; Adamson et al., 2010). It has shown excellent sensitivity (91%) and specificity (90%) in identifying CUD within clinical samples, with slightly lower but acceptable levels in community samples (Bruno et al., 2013). Despite this robust evidence base, screening tools validated in adults may not generalise to youth, given developmental differences in cognition, substance use patterns, and psychosocial context (Martin et al., 2006).

To date, no studies have evaluated the psychometric properties of the CUDIT-R in a purely youth sample. The present study aims to address this gap by examining the validity and reliability of the CUDIT-R in a clinical youth population with a history of cannabis use. We hypothesised that the CUDIT-R would demonstrate good psychometric performance and retain the unidimensional structure established in adult samples.

## Methods

### Design

The current study is a secondary analysis of data collected for a report examining the treatment outcomes in a youth therapeutic programme (Schroder et al., 2012). A longitudinal prospective design was utilised for the primary study to assess measures over a 12-month period. All procedures were fully approved by the Ministry of Health’s South Regional Ethics Committee (reference number: URA/05/05/052; Schroder et al., 2012).

### Sample

Participants were patients in the Odyssey House Christchurch Youth Day and Residential Alcohol and Other Drug (AOD) treatment programme, a single-centre specialist service in Christchurch, New Zealand. To be eligible for the study, participants had to enter treatment between 2006 and 2010, be aged 13–19 years, and meet criteria for one or more DSM-IV substance use disorder diagnoses. The study successfully enrolled 80 patients (Schroder et al., 2012). For the current study, patients who reported cannabis use within the preceding six months were eligible, resulting in a sample of 76 participants. Four patients were excluded due to no recent cannabis use. This sample size was deemed adequate as several studies outline that between 2 and 20 participants per item measure are required to undertake psychometric testing and confirmatory factor analysis (Kline, 2011; Morgado et al., 2018).

### Measures

In addition to the CUDIT-R, the following measures were used:

*Sociodemographic details* were obtained from each participant, including age, gender, and ethnicity.

To determine substance use diagnosis, *the Structured Clinical Interview for DSM-IV Axis I Disorders Patient Edition– Version 2.0* (SCID-I; Spitzer et al., 1988) was utilised to assess current and lifetime diagnoses of abuse and dependence for a variety of substances, including cannabis. The dependence and abuse symptoms were rated as “absent,” “subclinical” or “clinically present.”

The *Timeline Followback interview* (TLFB; Sobell & Sobell, 1992) is a structured interview and was utilised to calculate frequency and quantity of cannabis used throughout the previous 42 days. The TLFB has shown to have excellent reliability (Cronbach’s alpha = 0.79 to 0.98) and robust construct validity (Sobell et al., 1979; Stein et al., 2010).

### Procedure

Eligible patients were invited to partake in the treatment outcomes study by Odyssey staff and, if interested, gave written informed consent. Data was collected in the form of interviews conducted by trained researchers from the National Addiction Centre (NAC) at the University of Otago. The interviews were performed face-to-face in a private room to ensure privacy was maintained and took approximately two hours to complete. Throughout these interviews participants completed all measures. The interviews were conducted as close to the admission date to Odyssey House as possible and on average were completed within 15 days of admission. All participants were undergoing psychological treatment at the time of their interviews.

### Data Analysis

All statistical analyses were performed using both SPSS and AMOS statistical software packages (IBM Corp, 2017; Arbuckle, 2016). Descriptive statistics are reported to summarise the background characteristics of the sample and the item characteristics of the CUDIT-R. All the data was examined for normality using the skewness and kurtosis statistics and were found to be normally distributed (Brown, 2006).

To assess whether the CUDIT-R retained its single-factor structure in this novel population, a confirmatory factor analysis (CFA) was conducted using AMOS (Arbuckle, 2016), which is recommended for instruments with prior evidence of validity and reliability (Hurley et al., 1997). Data were analysed using CFA maximum likelihood estimation. Model fit was evaluated using multiple indices: the Chi-Square Test, Confirmatory Fit Index (CFI), Tucker-Lewis Index (TLI), and Root-Mean-Squared Residual (RMSEA). For smaller samples (*N* < 250), RMSEA may yield high type I error, so CFI and TLI were used (Hu & Bentler, 1998).

The psychometric properties of the CUDIT-R were also explored following previous CUDIT-R validation processes (Adamson et al., 2010) as well as Terwee’s best-practice guidance (Terwee et al., 2007). Cronbach’s alpha was used to assess the internal consistency reliability of the scale (Field, 2013). Each item of the CUDIT-R was assessed for both floor and ceiling effects, which are deemed to be present if 15% or more of the participants reach the highest or lowest possible score of an item (Terwee et al., 2007).

To assess discriminant validity, ROC analysis was used to determine optimal CUDIT-R cut-off scores for identifying cannabis dependence, as validated by the SCID. A ROC curve plots sensitivity against the false positive rate, with the Area Under the Curve (AUC) indicating test performance—values closer to 1.0 reflect better accuracy, while 0.5 suggests chance-level performance (Swets, 1992). Positive Predictive Value (PPV) and Negative Predictive Value (NPV) were also examined to evaluate diagnostic precision. Youden’s Index was applied to identify the best cut-off score, with values above 50% considered adequate for diagnostic utility (Youden, 1950). Additionally, independent t-tests compared CUDIT-R scores between those with and without a DSM-IV cannabis dependence diagnosis to determine if mean differences were statistically significant (Field, 2013). These combined analyses aimed to evaluate the effectiveness of the CUDIT-R in distinguishing individuals with cannabis dependence in a clinical adolescent sample.

Finally, concurrent validity was examined using bivariate correlations between CUDIT-R and a computed cannabis consumption score from the TLFB measure (i.e.: percentage days using x joints per day). To ensure that the observed relationship is not simply occurring due to the presence of the two consumption items, these items were removed to assess whether the TLFB measure correlates with the other domains of the CUDIT-R.

## Results

### Descriptive Statistics

The sample consisted of 76 participants aged 14–18 years (M = 16.3, SD = 1.1). *N* = 56 (74%) were male and *n* = 20 (26%) female. Participants identified as European (*N* = 38; 50%), Māori (*N* = 35; 46%), and other (*N* = 3; 4%). Fifty-eight (76%) were residential patients and 18 (24%) were day hospital patients. All (100%) reported cannabis use in the past six months and met DSM-IV criteria for cannabis abuse, except where excluded by cannabis dependence, which was present in 89% (*N* = 68). The average age of cannabis use onset was 11.9 years (SD = 2.0). Alcohol and other substance misuse were prevalent: 71% (*N* = 54) met criteria for alcohol dependence and 67% (*N* = 50) had more than one substance-use dependence, including cannabis. After cannabis and alcohol dependence, the most common DSM-IV diagnosis was stimulant dependence.

Total scores of the CUDIT-R ranged from 3 to 32 (*M* = 22.76, *SD* = 7.16). The computed consumption score of the TLFB data demonstrated that mean number of joints consumed on use days during the six weeks preceding interview ranged from 0 to 15.93 (*M* = 2.97; *SD* = 3.39). It is important to note that whilst all participants had used cannabis within the previous six months, some would have stopped prior to the TLFB assessment period of six weeks.

### Item Characteristics

The item-total correlation for item 8 was 0.104, with the remaining items ranging between 0.393 (item 4) to 0.678 (item 3). The items were assessed for skewness and kurtosis, the CUDIT-R item distributions had a skewness range between − 2.339 and − 0.132 and a kurtosis range between − 1.735 and 4.341. The values for both were deemed to be within the acceptable range (Brown, 2006). The performance of the full 8-item CUDIT-R is shown in supplementary material Table 1.

### Confirmatory Factor Analysis (CFA)

CFA was used to assess and confirm the one-factor structure of the CUDIT-R. The one-factor model showed a good fit, with χ² = 12 (df = 14) and RMSEA = 0.000 (CI: 0.000–0.079), indicating excellent fit. The CFI and TLI, which should exceed 0.95, were 1.000 and 1.037, respectively (Schreiber et al., 2006). Table 2 (see supplementary material) presents the factor loadings. Seven items loaded highly (above 0.4) and were highly significant (*p* <.001). Item 8 did not load with the others, indicating it did not align with the single factor measured by the other items in this population. Nevertheless, a one-dimensional approach with all 8-items was taken for the subsequent analyses. Although the empirically extracted factors were logical, the inclusion of a one-item factor was not practical and did not significantly enhance the scale’s validity. In addition, the present study aim was to assess the performance of the complete CUDIT-R within this novel population, rather than removing underperforming items.

### Internal Consistency

Internal consistency analysis demonstrated a Cronbach’s alpha score of 0.759, which falls within the recommended cut-off points for “good” reliability of 0.7 and 0.8 (Terwee et al., 2007).

### Floor and Ceiling Effects

Response distribution tended to be skewed towards the higher scores for each item, with all 8-items demonstrating ceiling effects. Four items also demonstrated floor effects, with more than 15% of the sample endorsing the lowest score.

### Concurrent Validity

CUDIT-R score was moderately correlated with a computed TLFB score of cannabis consumption, with Pearson correlations demonstrating *r* =.286, *p* <.005. The moderate correlation amongst the CUDIT-R and TLFB score persisted when the two items concerning cannabis consumption were removed from the analysis (*r* =.274, *p* <.005).

### Discriminant Validity

The AUC of the ROC curve was used to measure the general performance of the CUDIT-R in a youth clinical sample. The AUC was 0.96 (95% *CI* 0.89–1.0) demonstrating a 96% likelihood that a participant who had a SCID diagnosis of cannabis dependence would achieve a higher overall score on the CUDIT-R than a participant without a cannabis dependence diagnosis. The confidence intervals of CUDIT-R indicate excellent diagnostic accuracy (Swets, 1992). Sensitivity and specificity of the CUDIT-R, with feasible scores ranging from 0 to 32, alongside PPV and NPV are shown in supplementary material Table 3. An optimal solution appears with a CUDIT-R score of 12 or above (Youden index = 0.69), which identified 97.1% of participants diagnosed with a current cannabis use dependence at or above this level and 71.4% of participants without a current cannabis use dependence diagnosis scoring below this level.

An independent t-test was conducted to compare mean CUDIT-R scores across the presence or absence of the DSM-IV diagnostic criteria for cannabis dependence. Individuals with a cannabis use dependence scored higher on the CUDIT-R (*n* = 68, *M* = 24.25, *SD* = 5.74), than those without a cannabis use dependence diagnosis (*n* = 7, *M* = 9.71, *SD* = 5.85). The difference, −14.5 points (*CI*: −19.19– −9.44), was significant *(t* (73) = −6.37, *p* = < 0.001).

## Discussion

The present study aimed to assess the applicability of the Cannabis Use Disorders Identification Test-Revised (CUDIT-R) within a youth clinical sample characterised by high levels of cannabis use. Findings suggest the measure may not function identically in youth as it does in adults. While the one-factor structure found in adult samples showed adequate fit, item 8 (“considering cutting down use”) did not load as expected, indicating differing performance in this younger cohort. Although internal consistency was acceptable (Cronbach’s α = 0.76), the single-factor solution explained only 42.5% of the shared variance.

The dominant factor, which captured usage and adverse consequences (items 1–7), aligned with previous literature linking frequent youth cannabis use to negative outcomes (Volkow et al., 2016). However, item 8—measuring motivation to reduce use—formed a separate factor. This divergence may reflect unique motivational patterns in youth, as existing literature suggests that cannabis-related motivation differs in youth compared to adults (Lac & Luk, 2017; Pacheco-Colón et al., 2019). Youth may be less inclined to reduce cannabis use, particularly if co-occurring substance dependence is present and cannabis is perceived as less harmful than other drugs (Friese, 2017; UNODC, 2018). Item 7– measuring cannabis use in situations that could be physically hazardous such as driving– was found to be valid in this sample, which may be surprising given its focus. However, in New Zealand the legal age to drive is 16, and it may also be endorsed if participants used cannabis as passengers in vehicles driven by peers who were also using, broadening its relevance.

This variation in response highlights that youth may not engage with the CUDIT-R in the same way as adults. While this study did not aim to revise the scale, future research should explore whether structural adaptations are needed to optimise its use for younger populations. Notably, item 8 remains clinically valuable as it captures an important dimension of treatment planning: the individual’s readiness to change.

Overall, the psychometric performance of the CUDIT-R in this study was comparable to findings from adult samples, especially in terms of internal consistency and concurrent validity (Adamson et al., 2010; Schultz et al., 2019). However, response patterns showed ceiling effects, particularly for items assessing frequency and quantity of use, which is expected in heavy-using clinical populations. While such skew limits nuance in measuring severity, the CUDIT-R remains a screening tool rather than a diagnostic or severity index (Adamson et al., 2010). Thus, these effects are not a significant concern for its intended use (Allen, 2017).

Discriminant validity was supported, as CUDIT-R scores clearly distinguished participants with cannabis dependence from those without. However, the small number of non-dependent participants (*n* = 8) limited exploration of optimal cut-off scores for diagnostic accuracy. The sample’s clinical complexity and high disorder severity—alongside prevalent poly-drug use—further restrict generalisability to broader youth populations.

Several limitations should be noted. First, the sample was drawn from a treatment-engaged population between 2006 and 2010, limiting relevance to contemporary cohorts. Cannabis products, including potency, have significantly changed in the last 15 years. For example, potency is estimated to be higher in today’s market (ElSohly et al., 2016). Moreover, the CUDIT-R was developed and examined against DSM-IV (APA, 2013) criteria. The publication of the DSM-5 resulted in significant alterations to the classifications of CUDs, such as the removal of abuse and dependence criteria for an overall severity score (i.e.: mild, moderate or severe CUD; APA, 2013). Whilst the CUDIT-R has been validated for use alongside the updated classifications (Bruno et al., 2013; Schultz et al., 2019), it is vital that the practicality of measure is assessed in an adolescent clinical sample alongside the current diagnostic classification of CUDs. While sample size was sufficient for the conducted analyses, a larger cohort would reduce the risk of type-II error. Indeed, some methodologists recommend a sample of over 200 participants to undertake a Confirmatory Factor Analysis and other psychometric testing (White, 2022). Another limitation was the focus on cannabis dependence, rather than combining abuse and dependence for the ROC analysis. Whilst we did this to ensure stringency and clinical meaningful testing, it may have skewed the disability assessment, given that all participants met criteria for both presentations.

Analytically, test-retest reliability and predictive validity were not assessed. Future studies should evaluate the scale’s temporal stability and its ability to predict related constructs such as frequency of use. Furthermore, no qualitative data were gathered to assess face validity or item relevance for youth—a necessary step given the adult-oriented development of the scale. Comparison with alternative screening tools, such as the Cannabis Abuse Screening Test (CAST; Legleye et al., 2015), would also enhance the evaluation of criterion validity.

Despite these limitations, this study provides the first published evidence supporting the use of the CUDIT-R in an youth clinical sample. The scale effectively distinguished those with cannabis dependence and demonstrates clinical utility in identifying problematic use. Its brevity, ease of administration, and straightforward scoring make it a practical tool in busy clinical environments. Although screening tools are not substitutes for comprehensive assessment, the CUDIT-R can support clinicians in identifying CUDs among youth, much as it has been used in adult populations (Adamson et al., 2010; Annaheim et al., 2008). It may also inform treatment intensity and monitor outcomes, although this requires further validation.

In conclusion, this study offers initial support for the use of the CUDIT-R in youth clinical settings but suggests caution when interpreting results, particularly regarding motivation-related items. Further research in larger and more diverse youth samples is essential to evaluate and potentially refine the scale for this population.

## Supplementary Information

Below is the link to the electronic supplementary material.

Supplementary file1 (DOCX 20 kb)

## Data Availability

The data that supported the study’s findings are available from upon request.
