# Supplementary material for: Psychometric Performance of the Cannabis Use Disorders Identification Test– Revised (CUDIT-R) in an Youth Clinical Sample
Source: Community Ment Health J. 2025 Jul 23;61(8):1592–8. doi: 10.1007/s10597-025-01494-5 (PMC12647225; doi:10.1007/s10597-025-01494-5)
Supplement: Supplementary file 1 — Supplementary file1 (DOCX 20 kb) [file 10597_2025_1494_MOESM1_ESM.docx]

**Supplementary materials:**

Table 1: Item properties for CUDIT-R, n=76

|  | Corrected item-total correlation | Skewness | Kurtosis | Item mean | Item SD | Score distribution* (%) | | | | |
| --- | --- | --- | --- | --- | --- | --- | --- | --- | --- | --- |
|  |  |  |  |  |  | 0 | 1 | 2 | 3 | 4 |
| **Consumption** | | | | | | | | | | |
| *Item 1:* How often do you use cannabis? | 0.542 | -2.339 | 4.341 | 3.67 | 0.790 | 0 | 3.9 | 7.9 | 5.3 | 82.9 |
| *Item 2:* How many hours were you “stoned” on a typical day when you had been using cannabis? (Less than 1 through to 7 or more) | 0.441 | -0.924 | -0.620 | 3.17 | 1.088 | 0 | 11.8 | 15.8 | 15.8 | 56.6 |
| **Cannabis Problems (abuse)** | | | | | | | | | | |
| *Item 4:* How often during the past 6 months did you fail to do what was normally expected from you because of using cannabis? | 0.393 | -0.132 | -1.735 | 2.05 | 1.720 | 35.5 | 5.3 | 9.2 | 18.4 | 31.6 |
| *Item 7:* How often do you use cannabis in situations that could be physically hazardous, such as driving, operating machinery or caring for children? | 0.568 | -0.308 | -1.633 | 2.26 | 1.684 | 27.6 | 10.5 | 6.6 | 18.4 | 36.8 |
| **Dependence** | | | | | | | | | | |
| *Item 3:* How often during the past 6 months did you find that you were not able to stop using cannabis once you had started? | 0.678 | -0.827 | -1.108 | 2.71 | 1.680 | 23.7 | 3.9 | 3.9 | 14.5 | 53.9 |
| *Item 5:* How often in the past 6 months have you devoted a great deal of your time to getting, using or recovering from cannabis? | 0.542 | -1.762 | 1.685 | 3.32 | 1.278 | 7.9 | 6.6 | 2.6 | 11.8 | 71.1 |
| **Psychological Features** | | | | | | | | | | |
| *Item 6:* How often in the past 6 months have you had a problem with your memory or concentration after using cannabis? | 0.628 | -0.780 | -0.813 | 2.76 | 1.441 | 11.8 | 10.5 | 14.5 | 15.8 | 47.4 |
| *Item 8:* Have you ever thought about cutting down, or stopping, your use of cannabis? | 0.104 | -0.909 | -1.096 | 2.82 | 1.764 | 26.3 |  | 6.6 |  | 67.1 |
| **Total CUDIT-R Score** | - | -0.889 | 0.119 | 22.76 | 7.16 | - | - | - | - | - |

| Item 1 (frequency of use) | 0.57* | 0.60* |
| --- | --- | --- |
| Item 2 (hours “stoned”) | 0.72* | 0.55* |
| Item 3 (unable to stop once started) | 1.54* | 0.76* |
| Item 4 (role interference) | 1.00* | 0.48* |
| Item 5 (time devoted) | 0.99* | 0.65* |
| Item 6 (memory/concentration problems) | 1.17* | 0.67* |
| Item 7 (hazardous situations) | 1.54* | 0.68* |
| Item 8 (cutting down/stopping) | 0.32 | 0.15 |

**Table 2.** Standardised and unstandardised estimates of a one-factor model for the CUDIT-R

Note. All asterisk parameters significant, *p* < .001

**Table 3.** Identification of cannabis dependency disorder by CUDIT-R score, n = 76

| CUDIT-R score equal to or greater than | Positive Predictive Value | Negative Predictive Value | Sensitivity | Specificity |
| --- | --- | --- | --- | --- |
|  |  |  |  |  |
| 3 | 0.906 | 1.000 | 1.000 | 0.000 |
| 5 | 0.918 | 1.000 | 1.000 | 0.143 |
| 6 | 0.931 | 1.000 | 1.000 | 0.286 |
| 8 | 0.957 | 0.875 | 1.000 | 0.429 |
| 10 | 0.943 | 0.777 | 0.985 | 0.429 |
| 12^a^ | 0.970 | 0.700 | 0.971 | 0.714 |
| 13 | 0.970 | 0.636 | 0.956 | 0.714 |
| 14 | 0.969 | 0.583 | 0.941 | 0.714 |
| 15 | 0.983 | 0.500 | 0.926 | 0.857 |
| 16 | 0.983 | 0.466 | 0.897 | 0.857 |
| 17 | 0.983 | 0.388 | 0.882 | 0.857 |
| 18 | 0.982 | 0.368 | 0.838 | 0.857 |
| 20 | 0.982 | 0.333 | 0.824 | 0.857 |
| 21 | 1.000 | 0.291 | 0.779 | 1.000 |
| 22 | 1.000 | 0.269 | 0.735 | 1.000 |
| 23 | 1.000 | 0.218 | 0.706 | 1.000 |
| 24 | 1.000 | 0.189 | 0.618 | 1.000 |
| 25 | 1.000 | 0.162 | 0.544 | 1.000 |
| 26 | 1.000 | 0.159 | 0.456 | 1.000 |
| 27 | 1.000 | 0.140 | 0.441 | 1.000 |
| 28 | 1.000 | 0.118 | 0.353 | 1.000 |
| 29 | 1.000 | 0.111 | 0.221 | 1.000 |
| 30 | 1.000 | 0.107 | 0.162 | 1.000 |
| 31 | 1.000 | 0.102 | 0.132 | 1.000 |
| 32 | 1.000 | 0.094 | 0.088 | 1.000 |

^a^ Optimal combination of both sensitivity and specificity, based upon Youden’s index

* When 0.5 scores were identified from the analysis, they were rounded up to reflect the possible scores on the CUDIT-R.
